# Correction to “A multicenter prospective observational study of lymph node metastasis patterns and short‐term outcomes of extended lymphadenectomy in right‐sided colon cancer”

**DOI:** 10.1002/ags3.12797

**Published:** 2024-03-13

Tsukamoto S, Ouchi A, Komori K, Shiozawa M, Yasui M, Ohue M, et al. A multicenter prospective observational study of lymph node metastasis patterns and short‐term outcomes of extended lymphadenectomy in right‐sided colon cancer. *Ann Gastroenterol Surg*. 2023; 7: 940–948. https://doi.org/10.1002/ags3.12703

Subsequent to the issue publication, the authors added a supporting information file to the above article. This will help the readers understand the percentages shown in Figure 2.

The addition of the Supporting Information does not affect the above article.

